# Clinical and molecular epidemiology of enterovirus D68 from 2013 to 2020 in Shanghai

**DOI:** 10.1038/s41598-024-52226-w

**Published:** 2024-01-25

**Authors:** Fei Li, Rou-jian Lu, Yu-han Zhang, Peng Shi, Yuan-yun Ao, Lin-feng Cao, Yu-lan Zhang, Wen-jie Tan, Jun Shen

**Affiliations:** 1 Infectious Disease Department, Children’s Hospital of Fudan University, National Children’s Medical Center, Shanghai, China; 2 National Institute for Viral Disease Control and Prevention, China CDC, Beijing, China; 3 Statistics and Data Management Center, Children’s Hospital of Fudan University, National Children’s Medical Center, Shanghai, China; 4 Virology Department, Children’s Hospital of Fudan University, National Children’s Medical Center, Shanghai, China; 5 Wuhan Institute of Virology, Chinese Academy of Sciences, Wuhan, China

**Keywords:** Virology, Viral epidemiology

## Abstract

Enterovirus D68 (EV-D68) is an emerging pathogen that has caused outbreaks of severe respiratory disease worldwide, especially in children. We aim to investigate the prevalence and genetic characteristics of EV-D68 in children from Shanghai. Nasopharyngeal swab or bronchoalveolar lavage fluid samples collected from children hospitalized with community-acquired pneumonia were screened for EV-D68. Nine of 3997 samples were EV-D68-positive. Seven of nine positive samples were sequenced and submitted to GenBank. Based on partial polyprotein gene (3D) or complete sequence analysis, we found the seven strains belong to different clades and subclades, including three D1 (detected in 2013 and 2014), one D2 (2013), one D3 (2019), and two B3 (2014 and 2018). Overall, we show different clades and subclades of EV-D68 spread with low positive rates (0.2%) among children in Shanghai between 2013 and 2020. Amino acid mutations were found in the epitopes of the VP1 BC and DE loops and C-terminus; similarity analysis provided evidence for recombination as an important mechanism of genomic diversification. Both single nucleotide mutations and recombination play a role in evolution of EV-D68. Genetic instability within these clinical strains may indicate large outbreaks could occur following cumulative mutations.

## Introduction

Enterovirus D68 (EV-D68) is a single positive-stranded RNA virus, which is currently classified in the genus *Enterovirus* within the *Picornaviridae* family, and is known has 4 subspecies or clades (clade A to clade D) today^1^. Before 2014, there were few reported cases of EV-D68 infection globally. In August 2014, a global outbreak of EV-D68 was first observed, primarily affecting children, notably in the United States and Canada^2,3^. During the 2014 outbreak, over 2000 cases of EV-D68 infection were reported worldwide^2,4^. This event brought significant attention to EV-D68. Several countries and regions, including the United States, European Union, Japan, Canada, Australia, and Taiwan, initiated surveillance for EV-D68^5–7^. EV-D68 causes human respiratory disease and is widespread worldwide^8,9^. EV-D68 infection has occasionally been implicated in severe respiratory infections and central nervous system infections such as acute flaccid myelitis (AFM); however, no effective vaccines or antiviral treatments are currently available^5,10^. Recent studies provided comprehensive descriptions of how changes in the EV-D68 capsid antigens, host cell receptor usage, and neurotropism have increased the virulence of EV-D68, which has intensified global concern related to the serious threat EV-D68 poses to health^11,12^.

Sero-epidemiological studies have demonstrated a sustained, high prevalence of EV-D68 in the Chinese population, with varying rates among different age groups. Specifically, the seroprevalence rate was negatively correlated with age in infants (84.0% for 1-month-olds vs. 10.0% for 1-year-olds) and positively correlated in children (starting at 10% for 1-year-olds and reaching 92% for 15-year-olds)^13,14^. The situation is similar in other countries, especially as B3 subclade strains of EV-D68 have emerged as important pathogens that cause AFM and pediatric lung disease that requires supplemental oxygen. In the USA, New York observed a biennial upsurge from 2014 to 2018, characterized by subclades B1, B2, and the emergence of B3 strains causing severe respiratory illness and acute flaccid myelitis, with detection rates in the respiratory tract of 37.0%, 16.0%, and 29.0% in 2014, 2016, and 2018, respectively^7^. From July to November in 2018 to 2020, the positivity rate for EV-D68 detection was 10.8%, 0.2%, and 1.4%, respectively^15^. Additionally, at Johns Hopkins Health System of USA, in 2022, EV-D68 cases surged, mainly affecting children and adolescents. Similarly, China reported distinct EV-D68 genotypes prevailing from 2011 to 2018, notably A2 and B3, with evidence of recombination events^16–18^.

The circulation of enteroviruses, including EV-D68, since 2020 may have been limited by the widespread COVID-19 mitigation measures^19–21^. However, surveillance is necessary to detect possible re-outbreaks of enteroviruses such as EV-D68.

In this study, we retrospectively screened for EV-D68 in respiratory specimens from children hospitalized with community-acquired pneumonia (CAP) in our medical center over the past 7 years. Our objective was to characterize the clinical and genetic characteristics of EV-D68 circulating in Shanghai. This information will also contribute to the future establishment of surveillance networks, preparation of rapid clinical diagnostic capabilities, and development of vaccines or antiviral drugs based on the complete sequences published in this work.

## Materials and methods

### Study design and participants

Patients less than 18-years-old hospitalized with CAP at Children’s Hospital of Fudan University from June 1, 2013, to December 31, 2020, were eligible for inclusion in this retrospective study. One Nasopharyngeal swab (NPS) or bronchoalveolar lavage fluid (BALF) sample was collected from each patient within 48 h of admission. We excluded cases of aspiration pneumonia or nosocomial pneumonia. The criteria for the severity of CAP were based on the 2011 Infectious Diseases Society of America management guidelines for CAP^22^.

The clinical characteristics of all patients with CAP were retrospectively retrieved from the hospital’s medical record database. Neurological involvement was defined as meningitis, encephalitis, or AFM. We retrieved the results of routine tests for eight respiratory viruses—respiratory syncytial virus (RSV), influenza virus A and B (FLU A/B), parainfluenza virus 1–3 (PIV1-3), adenovirus (ADV), and human metapneumovirus (hMPV) by direct fluorescent antibody (DFA) assays in the clinical laboratory.

### EV-D68 detection and sequencing

For this study, viral RNA was extracted and purified using QIAamp Viral RNA Mini Kits (QIAGEN, Germany). A commercial fluorescence RT-PCR kit (Qiagen, Hilden, Germany) targeting the 5′ non-translated region (5′-NTR) of enteroviruses was used to simultaneously detect all enteroviruses, including EV-D68^23^. The complete genome sequence of EV-D68 was obtained by Sanger sequencing of multiple amplified nucleic acid fragments. A set of ten pairs of primers were designed to cover the entire genomic region of EV-D68 using overlapping amplicon products of approximately 500–1200 bp (Supplementary Table 1).

### Phylogenetic and recombination analysis

Phylogenetic analysis of the viral nucleotide sequences based on the polyprotein gene (3D) or the complete genomes of EV-D68 were performed using the neighbor-joining method with 1000 bootstrap replicates with the MEGA 5.0 program. Multiple amino acid sequence alignment analysis was performed using the DNAMAN 6.0 program. Genetic recombination analyses based on the complete genomes of EV-D68 strains were conducted using BootScan analysis in SimPlot 3.5.1 software, with the default settings of a window size of 200 bp, step size of 20 bp, 100 replicates, gap stripping, the distance model (Kimura), and tree model (neighbor-joining).

### Statistical analysis

Descriptive analysis was performed for the demographic and clinical characteristics of hospitalized children who tested positive for EV-D68 and eight other respiratory viruses. Continuous variables are presented as mean ± SD (standard deviation). Non-normally distributed variables are expressed as the median (interquartile range, IQR) and were compared using non-parametric tests. IBM SPSS Statistics 22.0 was used for data analysis.

### Ethics approval and consent to participate

The authors are accountable for all aspects of the work in ensuring that questions related to the accuracy or integrity of any part of the work are appropriately investigated and resolved. This study involved human participants and was reviewed and approved by the medical ethics committee of Children's Hospital of Fudan University (2020–208), with a waiver regarding informed consent. The study was conducted in accordance with the Declaration of Helsinki (as revised in 2013).

## Results

### Virus detection and clinical characteristics

Of the 3997 respiratory samples assessed (including 3751 NPS samples and 246 BALF samples), 43 (1.1%) samples were positive for enteroviruses, including nine (0.2%) samples that were positive for EV-D68. The demographic and clinical characteristics of these EV-D68-positive cases are presented in Table 1.

**Table 1:** Demographics and clinical characteristics of EV-D68 positive cases.

Case	Gender	Age (year)	Collecting date	Specimen type	Diagnosis	Underlying disease	Fever (°C)	Other symptoms	Co-detected	Outcome	Strain	Clade
1	Male	0.9	2013.08	NPS	Pneumonia	None	39.0	Cough, wheezing	hMPV, Mp	Cured	KU242688	D1^a^
2	Female	1.5	2013.09	NPS	Pneumonia	Asthma	No	Cough, wheezing	None	Cured	KU242684	D1^a^
3	Male	3	2013.11	NPS	Pneumonia	None	No	Cough, wheezing, cyanosis	Mp	Cured	KU242689	D2^a^
4	Male	0.5	2014.03	NPS	Pneumonia	Congenital heart disease, Premature	No	Cough	RSV *S. aureus*	Cured	KU242690	D1^a^
5	Male	0.4	2014.07	NPS	Pneumonia	Agranulocytosis	39.6	Cough	PIV3, *P. aeruginosa*	Cured	MW697454	B3^b^
6	Male	1.5	2018.07	NPS	Pneumonia	None	38.7	Cough, diarrhea	PIV3	Cured	None	None
7	Female	1.2	2018.07	NPS	Pneumonia	None	No	Cough, wheezing	RV, *B. pertussis*	Cured	MW697453	B3^b^
8	Male	5.2	2019.11	NPS	Pneumonia	None	39.0	Cough	RV, Mtb	Cured	MW697455	D3^b^
9	Male	0.8	2020.09	NPS	Pneumonia	Congenital heart disease, hypogammaglobulinemia, epilepsy	No	Cough, wheezing	None	Cured	None	None

*NPS* nasopharyngeal swab, *hMPV* human metapneumovirus, *Mp* mycoplasma pneumoniae, *RSV* respiratory syncytial virus, *S. aureus* *Staphylococcus aureus*, *PIV3* *Parainfluenza virus type 3*, *P. aeruginosa* Pseudomonas aeruginosa, *RV* *Rhinovirus*, *B. pertussis* Bordetella pertussis, *Mtb* mycobacterium tuberculosis.

^a^Genotyping based on partial polyprotein gene (3D) sequence.

^b^Genotyping based on complete gene sequence.

All nine EV-D68-positive samples were NPS collected from children without severe CAP. In 2013, there were 3 cases, followed by 2 cases in 2014, 2 cases in 2018, 1 case in 2019, and 1 case in 2020. None of these nine cases had neurological involvement, although fever and wheezing were present in most cases. The median age of the nine EV-D68-positive children was 1.3 years (IQR: 0.4–5.2), seven (77.8%) of these children were less than 1-year-old. Additionally, routine analysis conducted by the clinical laboratory at the time of hospitalization reported that seven out of nine EV-D68-positive samples (77.8%) also tested positive for other respiratory pathogens (Table 1).

### Sequencing and phylogenetic analyses

Seven of the nine EV-D68 strains were sequenced; these sequences have been submitted to GenBank (KU242684, KU242688-90 and MW697453-55), including four partial genomes based on the polyprotein gene (3D) and three complete genomes (Supplementary Table 2). We also identified the locations of the main genes in the complete genomes. For example, the VP1 gene sequence was located at 2337‒3263 nt in the MW697453 strain and at 2348‒3274 nt in the MW697454 strain (Supplementary Table 3).

**Figure 1:**
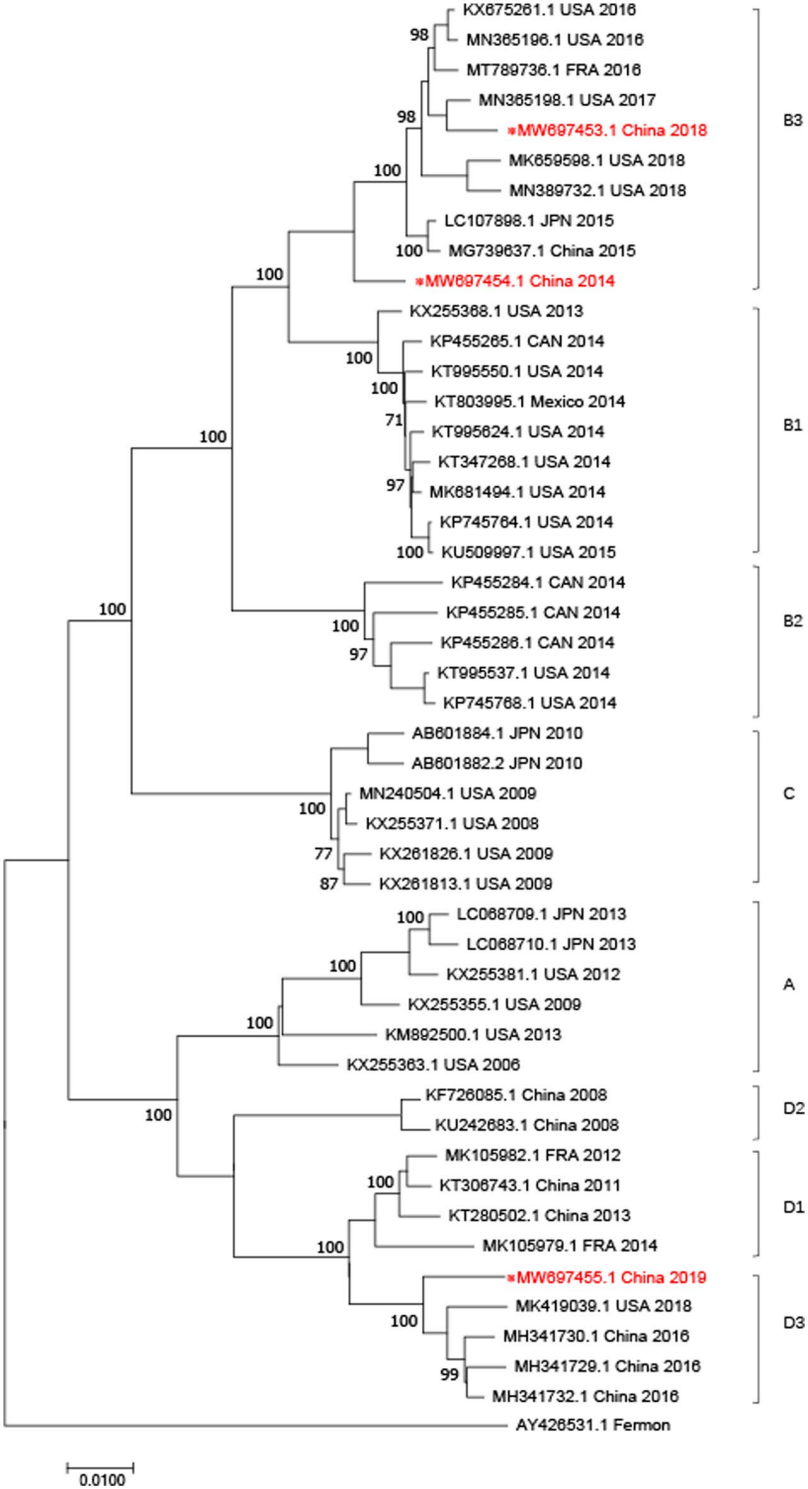
Phylogenetic analysis of EV-D68 based on the complete genomes. Three strains (in red) found in this study and other selected EV-D68 strains were analyzed by the Neighbor-Joining method with 1000 bootstrap replicates using the MEGA 5.0 program. The percentage of bootstrap values (percentage of 1000 replicate datasets) over 70% are shown at the nodes of the trees. Numbers on the nodes represent bootstrap support. ※: Strains submitted to the GenBank in this study.

**Figure 2:**
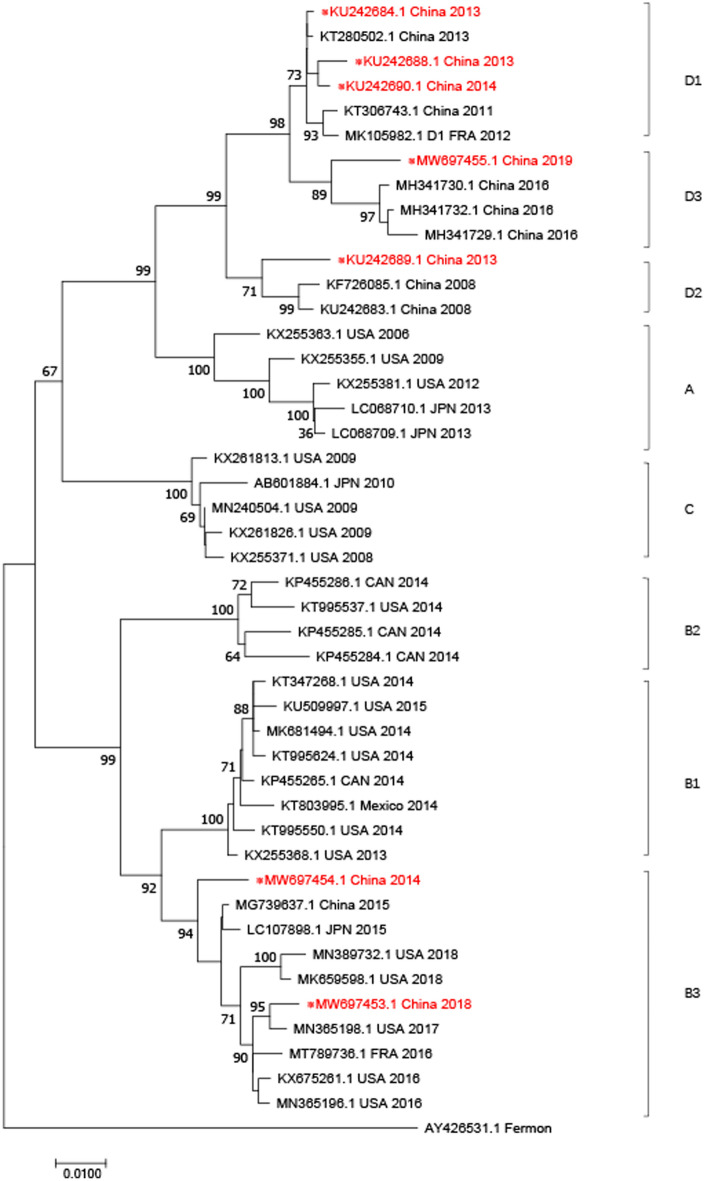
Phylogenetic analysis of EV-D68 based on the 3D gene (located between 5281 and 7261 nt of the polyprotein). The phylogenetic trees were generated by using the neighbor-joining method based on the Kimura two-parameter model with 1000 replicates.

We also performed complete genome and 3D genome sequence-based evolutionary analyses. The phylogenetic tree based on the three complete genomes showed that two strains detected in 2014 and 2018 belong to subclade B3, while the 2019 strain belongs to subclade D3 (Fig. 1). The phylogenetic tree based on the seven partial 3D genome sequences revealed a diversity of subclades (three D1, one D2, two B3, and one D3); however, the three strains with complete genomes were in the same subclades on both trees (Fig. 2).

### Amino acid homology analyses

Next, we analyzed amino acid homology in order to assess the possible impact of genetic diversity within these clinical strains. Assembly of the amino acid sequences of the three strains with complete sequences (MW697453, MW697454, and MW697455) revealed these strains shared 95.5–99.8% identity with 17 other representative EV-D68 strains (including the Fermon strain) in GenBank. Moreover, the differences in the amino acid sequence homology of the three strains with complete sequences identified in our study were less than 4%. Moreover, the average difference in the amino acid identities between all 20 strains was less than 5%, indicating that the amino acid sequences of different EV-D68 genotypes are relatively stable. Both MW697453 and MW697454 belong to subclade B3, and the differences in the amino acid homology between these two strains and the KX675261 strain, which was isolated from a case of AFM in March 2016 in America, were only 0.2% and 0.5%, respectively. Moreover, the maximum amino acid homology differences between the three D3 subclade strains identified in our studies (MW697455 isolated in this study, and the strains of MH341730 and MH341732, which were isolated in Beijing, China, in September and October 2016) were 0.7%, indicating that the amino acid homology differences between strains from the same genotype of EV-D68 are almost negligible (Table 2).

**Table 2:** Amino acid sequence homology analysis in the coding region of EV-D68.

Subtypes	Representative strains	AY426531	KM892500	KX255381	KP745764	KT995550	KP455285	KP745768	KX675261	MG739637	AB601882	KX261826	KT306743	MK105979	KF726085	KU242683	MH341730	MH341732	MW697453	MW697454	MW697455
Fermon	AY426531																				
	KM892500	0.045																			
A	KX255381	0.044	0.011																		
	KP745764	0.042	0.031	0.030																	
B1	KT995550	0.041	0.029	0.029	0.001																
	KP455285	0.039	0.027	0.026	0.015	0.014															
B2	KP745768	0.040	0.028	0.027	0.015	0.014	0.004														
	KX675261	0.037	0.030	0.028	0.014	0.013	0.011	0.011													
B3	MG739637	0.038	0.029	0.027	0.014	0.012	0.011	0.011	0.001												
	AB601882	0.041	0.026	0.026	0.022	0.020	0.015	0.016	0.019	0.019											
C	KX261826	0.038	0.023	0.023	0.020	0.019	0.015	0.016	0.018	0.018	0.005										
	KT306743	0.043	0.019	0.020	0.028	0.027	0.026	0.027	0.027	0.027	0.027	0.026									
D1	MK105979	0.045	0.022	0.022	0.031	0.029	0.028	0.027	0.028	0.027	0.031	0.029	0.007								
	KF726085	0.046	0.019	0.022	0.032	0.031	0.027	0.028	0.029	0.029	0.027	0.025	0.013	0.016							
D2	KU242683	0.044	0.018	0.021	0.030	0.029	0.025	0.026	0.027	0.027	0.025	0.023	0.012	0.015	0.004						
	MH341730	0.045	0.024	0.026	0.033	0.032	0.031	0.032	0.032	0.032	0.032	0.031	0.008	0.011	0.021	0.020					
D3	MH341732	0.042	0.022	0.024	0.032	0.030	0.030	0.031	0.031	0.030	0.031	0.029	0.006	0.010	0.019	0.018	0.003				
B3	MW697453	0.038	0.029	0.026	0.016	0.014	0.011	0.011	0.002	0.003	0.019	0.018	0.027	0.026	0.028	0.027	0.032	0.031			
B3	MW697454	0.038	0.029	0.027	0.013	0.011	0.011	0.011	0.005	0.005	0.018	0.016	0.026	0.028	0.030	0.028	0.031	0.029	0.007		
D3	MW697455	0.044	0.024	0.026	0.035	0.033	0.032	0.032	0.032	0.031	0.035	0.033	0.010	0.012	0.020	0.019	0.007	0.005	0.031	0.032	

The numbers in the table represent the differences in amino acid homology between these two strains, where smaller values indicate lesser differences. A value of 0.05 corresponds to a 5% difference in their percentage of the homology analysis.

### VP1 amino acid sequence analyses

We further analyzed the highly variable VP1 amino acid sequence that encodes the main receptor binding region. Within the motifs corresponding to the VP1 BC, DE, and GH loops and C-terminus, we found that MW697453 (B3) contained one unique substitution (E95T in the BC loop) and MW697455 (D3) had two unique substitutions (E95G and A96E in the BC loop) compared to 18 other EV-D68 strains. Moreover, the MW697453 (B3) and MW697454 (B3) strains contained N90D, T98A, and M148V substitutions in the BC and DE loops; these changes were present in all of the B clade strains within the 20 strains. In addition, the substitutions N140G, S143N, N145S, I187V, D297E, and N305D were found in the DE and GH loops and C-terminus of all strains belonging to the D clade, including our MW697455 (D3) strain (Table 3).

**Table 3:**
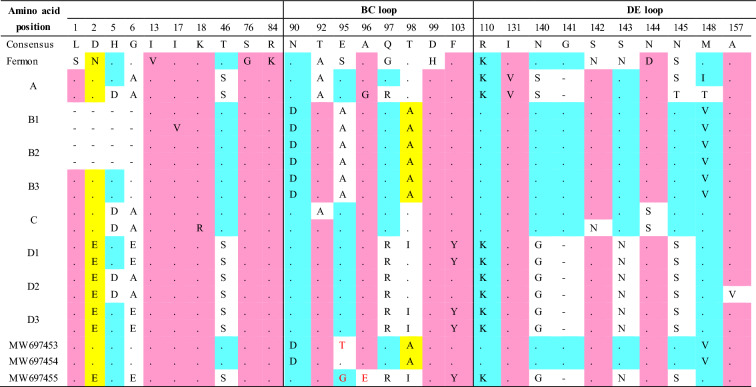

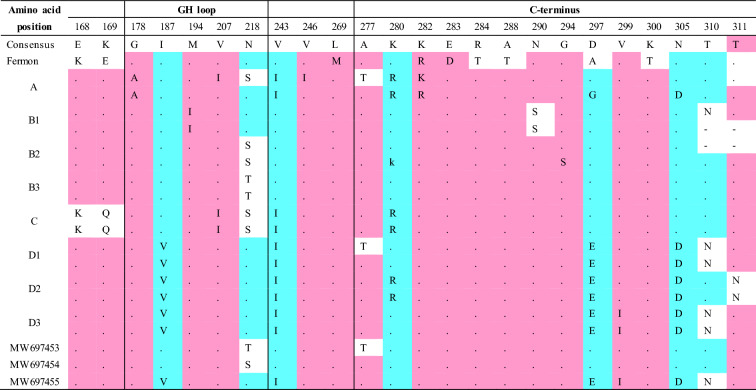
Amino acid sequence alignment of capsid protein VP1. Amino acid sequences encode the protein loops of capsid protein VP1 were highlighted by solid red lines.

(–): deleted sequences, (.): conserved sequences. Amino acid sequences homology: < 33%, in white; ≥ 33%, in yellow; ≥ 50%, in blue; ≥ 75%, in red; and 100% were not showed.

### Recombination analyses

**Figure 3:**
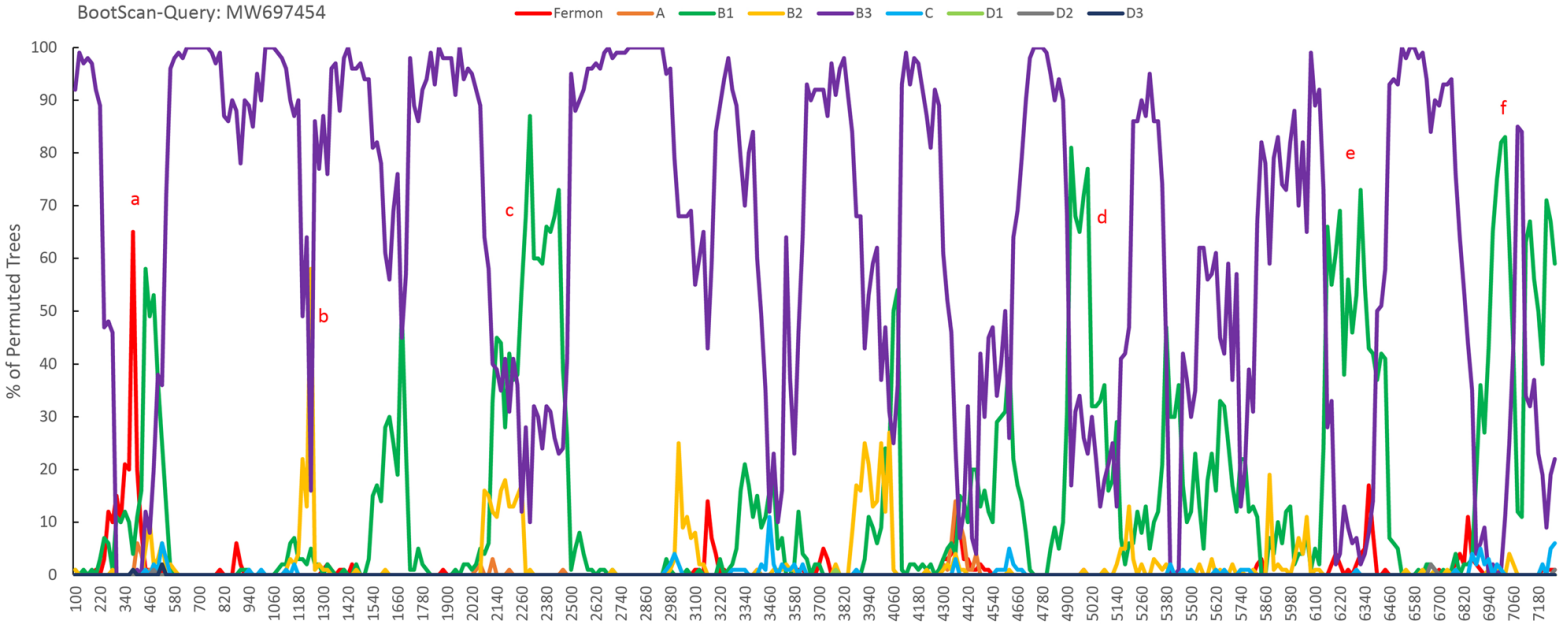
Genetic recombination analyses based on the complete genomes of EV-D68 strains. BootScan analysis. MW697454 was used as the query sequence and compared with nine other selected strains of EV-D68. The default settings of SimPlot 3.5.1 software were used: window size 200 bp, step size 20 bp, 100 replicates, gap stripping, distance model (Kimura), and tree model (neighbor-joining). Similarity plot of the complete genomes of MW697454 (B3), MW697453 (B3), and MW697455 (D3) with the sequences of other nine other strains in GenBank (AY426531, KM892500, KP745764, KP745768, KX675261, AB601882, KT306743, KF726085, and MH341732). (**a**–**f)** Potential positions of genomic recombination.

We observed recombination may frequently occur between the clades or subclades of the EV-D68 strains. Similarity plots and BootScan analysis using SimPlot 3.5.1 software suggested recombination events potentially occurred between EV-D68 A-D clades or subclades (Fig. 3). Compared to nine other selected EV-D68 strains available in GenBank—AY426531 (Fermon, A), KM892500 (A), KP745764 (B1), KP745768 (B2), KX675261 (B3), AB601882 (C), KT306743 (D1), KF726085 (D2), and MH341732 (D3)—the sequence of MW697454 (B3) identified in this study exhibited potential genome recombination with KP745764 (B1) in the 420–500, 2140–2480, 4920–5120, 6160–6400, and 6880–7260 nucleotide regions, and with KP745768 (B2) in nucleotides 1200–1260, respectively. KP745764 and KP745768 were isolated in September 2014 in the USA during an EV-D68 outbreak in North America. Moreover, the MW697453 (B3) strain potentially underwent genome recombination with KP745764 (B1) in the nucleotide regions 300–500 and 5100–5160. MW697455 (D3), exhibited potential recombination with KM892500 (A) in the 400–520 nucleotide region, with KT306743 (D1) in nucleotide regions 980–1020 and 6420–6460, with Fermon (A) in nucleotide region 1860–1940, and with KF726085 (D2) in nucleotide regions 5000–5160 and 6100–6180.

## Discussion

In this work, we demonstrated the sustained prevalence of EV-D68 among children in Shanghai from 2013 to 2020. The virus spread quietly among children in Shanghai during this period, without causing an outbreak or any severe cases, even in the first year of the SARS-CoV2 outbreak. One isolate of EV-D68 was detected in 2020 in this study. This finding is reasonable, as our previous study found the prevalence of respiratory and gastrointestinal viruses decreased significantly after implementation of the lockdown during the COVID-19 outbreak in Shanghai^19^. It remains to be seen whether the prevalence of EV-D68 increased when the community reopened, as reported in similar studies in European countries^21^.

Eight of the nine EV-D68-positive specimens identified in this study were collected between July and November. The detection rates of several common respiratory viruses in these specimens exhibited an obviously seasonal distribution in Shanghai, even though the detection rates of these viruses dropped sharply in 2020, as mentioned above (Table 1)^19,24^. None of the EV-D68-positive cases in this study had severe respiratory infections or neurological impairment; furthermore, most positive cases were less than 2-years-old and only half exhibited fever or wheezing, in agreement with previous reports^25,26^. Moreover, seven of the nine EV-D68-positive cases were also positive for other pathogens, including respiratory viruses and bacteria, as reported by the clinical microbiology laboratory. The co-detected pathogens were similar to previous studies conducted in Shanghai and Guangdong China^19,25–27^.

Hospital-based surveillance provides a useful platform for early detection of potential outbreaks and molecular characterization of emerging EV-D68 strains with higher virulence. The predominant EV-D68 clades may have shifted from clades A and C to clades B and D, and clades B and D have expanded worldwide^28–30^. We do not yet fully understand the dynamics of these changes or the significance of this viral evolution, nor can we predict the outcomes of EV-D68 infection based on the viral sequences or genotypes^31^. However, subclade B1 was most prevalent and associated with neurological complications in a 2014 outbreak, and the subclade B1 strains seem to have originated in Asia^16,32^. Since 2016, subclade B3 and D1 have become predominant in both the United States and European countries^33,34^. The B3 subclade strains were first identified in Taiwan and mainland China in 2014 and share a common ancestor with subclade B1^16,35^. Interestingly, two strains with complete sequences, MW697454 which was collected in 2014 and MW697453 identified in our study in 2018, both belong to the B3 subclade. We previously reported one strain collected in 2008 in Beijing named BJ24 (KU242683) belongs to subclade D1 based on complete gene sequence analysis. In this study, two strains (KU242684 and KU242688) that were collected in 2013 in Shanghai also belong to D1 based on 3D sequence analysis. This indicates that D1 subclade strains were already prevalent in China during the period from 2008 to 2013, before the B3 and D1 subclade strains were found to be dominant in the United States and Europe. Moreover, this study also detected one D2 subclade (KU242689) strain from the samples collected in 2013 in Shanghai based on 3D sequence analysis. Additional D3 subclade strains were detected in Beijing in 2016^30^. Several years later, we found another D3 strain (MW697455) in a sample collected in 2019 in Shanghai based on complete sequence analysis. This finding suggests that sustained local circulation of the D3 subclade strains may have occurred from 2016 to 2019. Thus, following the emergence of the D1 and B3 strains in China and their worldwide spread, we suspect that the D3 subclade strains detected in Shanghai will soon be detected or are already circulating in the United States and Europe.

EV-D68 has undergone significant genetic changes since the original identification of the four prototypical strains (Fermon, Franklin, Robinson, and Rhyne). These changes are predominately single nucleotide mutations that occur as random events during the course of viral replication. Such changes can lead to the emergence of an outbreak of severe cases due to a sudden increase in the pathogenicity of a virus. In this study, the divergence between the complete amino acid sequences of different EV-D68 clade or subclade strains (including the Fermon strain) was less than 5%. This result is well below the 85% amino acid homology threshold used to identify different species or genotypes of enteroviruses. In addition, the three strains for which we obtained complete genome sequences (MW697453, MW697454, and MW697455) in this study shared amino acid identities of 95.6–99.6%.

The divergence in the EV-D68 nucleotide or amino acid sequences between strains is mainly due to cumulative single nucleotide mutations, region deletion, and recombination. The most important cause of genetic diversity is a variety of changes to the amino acid sequences in the capsid region—including the capsid protein VP1, which contributes to the antigenicity of the virus and virus-host cell attachment^36,37^. Based on the 3D structures of EV-D68, the VP1 BC loop and VP1 DE loop are thought to be antigenic epitopes^38,39^. We found MW697453 (B3) had one unique substitution (E95T in the BC loop) and MW697455 (D3) had two unique substitutions (E95G and A96E in the BC loop) compared to 18 other EV-D68 strains. Codons 95 and 96 in the BC loop of EV-D68 VP1 play crucial roles in the evolutionary divergence of EV-D68 clades, subclades, and epidemics^40^. Moreover, the MW697453 (B3) and MW697454 (B3) strains contained the N90D, T98A, and M148V substitutions in the VP1 BC or DE loops, and these substitutions are present in all of the B clade strains within the 20 EV-D68 strains assessed in this study. However, the mechanisms underlying such substitutions remain unknown. All strains belonging to the D clade identified in this study, including the MW697455 (D3) strain, exhibit the N140G, S143N, N145S, I187V, D297E, and N305D substitutions in the VP1 DE and GH loops and the C-terminus. The presence of these substitutions in the D clade viruses is an interesting phenomenon that deserves further study. The I187V substitution in the VP1 GH loop affects the floor of the “canyon” on the surface of EV-D68 where the virus attaches to cellular receptors, such as the sialic acid receptor^41,42^. Lau et al.^43^ reported that amino acid residue 140 within the DE loop of EV-D68 VP1 was under potential positive selection in strains found in Hong Kong.

Similarity plots showed that the MW697453 strain (belonging to B3) potentially underwent genomic recombination in both the capsid genomic regions and non-capsid genomic regions with the B3 (KX675261) and B1 (KP745764) subclade strains. Genetic exchange between EV-D68 clades or subclades and other strains of enteroviruses probably results from their common tissue tropisms^44^. The KP745764 strain was isolated in September 2014 in the USA during the 2014 North American EV-D68 outbreak. Based on nucleotide identity analysis, researchers have speculated the strains that caused the North American EV-D68 epidemic in 2014 may have originated from strains that were circulating in Asia a few years previously. Therefore, it is reasonable to hypothesize that MW697453 might have undergone potential genome recombination with the ancestral strains of KP745764 prevalent in Asia.

This study has some limitations. First, we only enrolled patients hospitalized with CAP, who presumably had more severe symptoms than outpatients or patients who did not seek medical care. In addition, samples were not collected from cases with acute upper respiratory tract symptoms, and the number of positive samples was limited in our study. Therefore, further epidemiological studies, involving a larger number of cases, are crucial to comprehensively assess the seasonality, epidemiology, and disease impact of EV-D68 within the population. Second, we did not screen cerebrospinal fluid samples from cases with nervous system involvement, such as AFM or encephalitis, for EV-D68 at our hospital. Finally, we were unable to culture any of the strains in cell lines to evaluate the cell tropism of these clinical strains, including their potential infectivity towards nerve cells.

## Conclusion

In summary, despite low rate of detection of EV-D68 during the past 7 years, the clinical strains detected in different years in this study belonged to different clades or subclades. Moreover, potentially at least two different molecular evolution mechanisms underlie the emergence of these EV-D68 strains, including single nucleotide mutations and recombination between clades or subclades.

### Supplementary Information

Supplementary Table 1.

Supplementary Table 2.

Supplementary Table 3.

## Data Availability

The datasets generated during and/or analysed during the current study are not publicly available due to the data belong to the hospital database but are available from the corresponding author on reasonable request.

## Abbreviations

EV-D68: Enterovirus D68
NPS: Nasopharyngeal swab
BALF: Bronchoalveolar lavage fluid
CAP: Community-acquired pneumonia
AFM: Acute flaccid myelitis
RSV: Respiratory syncytial virus
FLU A/B: Influenza virus A and B
PIV1-3: Parainfluenza virus 1–3
ADV: Adenovirus
hMPV: Human metapneumovirus
DFA: Direct fluorescent antibody
5’-NTR: 5’ Non-translated region
IQR: Interquartile range
