# Supplementary material for: Clinical and molecular epidemiology of enterovirus D68 from 2013 to 2020 in Shanghai
Source: Sci Rep. 2024 Jan 25;14:2161. doi: 10.1038/s41598-024-52226-w (PMC10810781; doi:10.1038/s41598-024-52226-w)
Supplement: Supplementary file 1 — Supplementary Table 1. [file 41598_2024_52226_MOESM1_ESM.docx]

**Supplementary Table 1.** Universal Primers for complete genome amplification of EV-D68.

| Location | Name of primers | Primer sequences (5’-3’) |
| --- | --- | --- |
| 1-511bp | 1U21  1L20 | TTAAAACAGCYYTGGGGTTGT  CGGACTTGCGCRTTACGACA |
| 221-1157bp | 2U20  2L21 | AYYTATCCGTTATCCGCTAT  ATTYAGYGCATCRGGTARTTT |
| 1022-2075bp | 3U21  3L17 | GGTGAATGGCCYAATTAYTTA  CTGGTTGTYGGRCATGA |
| 1978-3004bp | 4U21  4L19 | YACYCATTGGTCTGGATCYCT  ACCRYTTTTCTCAAATCCR |
| 2885-3906bp | 5U21  5L19 | CAYTGGCAGTCAGSYAGTAAT  AYYAARTCYTCYGARTTTC |
| 3898-4744bp | 6U21  6L21 | TGTAATYAGAAAYTCRGARGA  TCAAATTTRAATCTRCGTGAY |
| 4449-5530bp | 7U21  7L21 | ATTTRATTGCYAGRGCTATCA  GTCAAGTCTCTRAGHGCACAY |
| 5312-6254bp | 8U18  8L21 | CCTAAACCYAARGTACCC  YCTTTTCTTCTTYCCYTGYAG |
| 5944-6862bp | 9U18  9L21 | CGCACCGGCAAAGACTAA  ATAACRTCATCYCCATAKGCW |
| 6690-7319bp | 10U19  10L21 | ATYTGTGYCAYTCRGTRCA  CCAAKTRRMCAAAATTTACCT |
