# Supplementary material for: Clinical and molecular epidemiology of enterovirus D68 from 2013 to 2020 in Shanghai
Source: Sci Rep. 2024 Jan 25;14:2161. doi: 10.1038/s41598-024-52226-w (PMC10810781; doi:10.1038/s41598-024-52226-w)
Supplement: Supplementary file 2 — Supplementary Table 2. [file 41598_2024_52226_MOESM2_ESM.docx]

**Supplementary Table 2.** The information of seven EV-D68 strains submitted to the GenBank in this research.

| Accession number | Genome | Length | Collection Date |
| --- | --- | --- | --- |
| KU242684 | partial genome | 7343bp | Sep-2013 |
| KU242688 | partial genome | 890bp | Aug-2013 |
| KU242689 | partial genome | 890bp | Nov-2013 |
| KU242690 | partial genome | 890bp | Mar-2014 |
| MW697453 | complete genome | 7309bp | Jul-2018 |
| MW697454 | complete genome | 7258bp | Jul-2014 |
| MW697455 | complete genome | 7304bp | Nov-2019 |
