# Supplementary material for: Clinical and molecular epidemiology of enterovirus D68 from 2013 to 2020 in Shanghai
Source: Sci Rep. 2024 Jan 25;14:2161. doi: 10.1038/s41598-024-52226-w (PMC10810781; doi:10.1038/s41598-024-52226-w)
Supplement: Supplementary file 3 — Supplementary Table 3. [file 41598_2024_52226_MOESM3_ESM.docx]

**Supplementary Table 3.** Gene locations in nucleotide sequences of three EV-D68 strains.

| Gene | MW697453 | MW697454 | MW697455 |
| --- | --- | --- | --- |
| 5’UTR | 1-680 | 1-691 | 1-682 |
| VP4 | 681-887 | 692-898 | 683-889 |
| VP2 | 888-1631 | 899-1642 | 890-1633 |
| VP3 | 1632-2336 | 1643-2347 | 1634-2338 |
| VP1 | 2337-3263 | 2348-3274 | 2339-3268 |
| 2a to 3d | 3264-7244 | 3275-7258 | 3269-7249 |
| 3’UTR | 7245-7258 | 7259-7309 | 7250-7304 |

MW697453：GenBank：<https://www.ncbi.nlm.nih.gov/nuccore/MW697453>

MW697454：GenBank：<https://www.ncbi.nlm.nih.gov/nuccore/MW697454>

MW697455：GenBank：<https://www.ncbi.nlm.nih.gov/nuccore/MW697455>
